# Burden from Study Questionnaire on Patient Fatigue in Qualitative Congestive Heart Failure Research

**DOI:** 10.3390/jcdd11040096

**Published:** 2024-03-24

**Authors:** Pupalan Iyngkaran, Wania Usmani, Zahra Bahmani, Fahad Hanna

**Affiliations:** 1 Department of Health and Education, Torrens University Australia, Melbourne, VIC 3000, Australia; pupalan.iyngkaran@student.torrens.edu.au (P.I.); wania.usmani@health.torrens.edu.au (W.U.); 2 HeartWest, Hoppers Crossing, VIC 3029, Australia; zahra.b77@outlook.com; 3 Public Health Program, Department of Health and Education, Torrens University Australia, Melbourne, VIC 3000, Australia

**Keywords:** cardiovascular disease, chronic disease management, health data, congestive heart failure, guidelines, processes of care, multidisciplinary care

## Abstract

Mixed methods research forms the backbone of translational research methodologies. Qualitative research and subjective data lead to hypothesis generation and ideas that are then proven via quantitative methodologies and gathering objective data. In this vein, clinical trials that generate subjective data may have limitations, when they are not followed through with quantitative data, in terms of their ability to be considered gold standard evidence and inform guidelines and clinical management. However, since many research methods utilise qualitative tools, an initial factor is that such tools can create a burden on patients and researchers. In addition, the quantity of data and its storage contributes to noise and quality issues for its primary and post hoc use. This paper discusses the issue of the burden of subjective data collected and fatigue in the context of congestive heart failure (CHF) research. The CHF population has a high baseline morbidity, so no doubt the focus should be on the content; however, the lengths of the instruments are a product of their vigorous validation processes. Nonetheless, as an important source of hypothesis generation, if a choice of follow-up qualitative assessment is required for a clinical trial, shorter versions of the questionnaire should be used, without compromising the data collection requirements; otherwise, we need to invest in this area and find suitable solutions

## 1. Introduction

With the abundance of multicentre randomised controlled trials referenced in congestive heart failure (CHF) guidelines, it is of interest to note that global reductions in mortality and morbidity outcomes have been patchy [1,2]. Considering the selective nature of the trials with stringent inclusion criteria, a way to attain a more consistent outcome is to study a more diverse treated population and, consequently, reflect on those who exhibit poorer outcomes. The methodology for such research is well established in phase IV or translational research methodologies. This type of research is often in the form of audits, which are informative and hypothesis-generating, should novel observations be recorded. This type of research also has foundations in the mixed methods research methodology (MMR). In the first section, we discuss the role of data from community hubs, which are at the forefront of ambulatory clinical translational models [3]. Randomised controlled trials (RCT) are rigid, and the trial elements are limited to intervention and objective (quantitative) outcomes. Their findings are highly translatable to the question and the population studied [4,5,6]. Hence, these trials form the gold-standard evidence that is the backbone of evidence-based medicine (EBM) and guideline-derived medical therapies (GDMT) in clinical guidelines. Furthermore, in this paper (part 2), the two relevant critical elements are disease complexity (e.g., multimorbidity) and layered health services (e.g., multidisciplinary teams), which are required to manage chronic cardiovascular diseases (CVD). The real-world scenarios are not frequently reflected in clinical trials, and, therefore, the means to translate proven evidence and complex care at the population level are needed.

There is, however, robust post-translational evidence for CHF. Generic cardiac and, more specifically, CHF rehabilitation are established in evidence, clinically translated by utilising multidisciplinary care to support improved outcomes [7,8,9], including in the elderly [10]. Care, with similar foundations as cardiac rehabilitation, is routinely advocated in clinical guidelines as a translational tool to enhance multidisciplinary care, following gold-standard evidence in trials and clinical translation [11]. It is important to understand that these management tools are not evidence of therapies as such but measures to collate guideline evidence and deliver it in an organised fashion. There are also failures in multidisciplinary approaches, where the collection of evidence has stalled, and guideline recommendations have even been demoted. Self-management programs in CVD and CHF have been downgraded from performance to quality measures [12]. Importantly, this concept requires a mixed methods approach to generate new ideas. Thus, RCTs are the engine for proof, while MMR is the machinery used for innovation, discovery and creating a combined approach to deliver the findings. In particular, subjective questionnaires are an important component of this methodology. In this second section, the authors aim to explore the gaps and avenues needing to be innovated in this area.

## 2. Mixed Method Research in Translating CVD and CHF Guidelines

MMR is a research methodology that cumulates, investigates, and interprets data within a study or program of research. MMR utilises both quantitative and qualitative methods for investigating diseases such as CHF and CVD [13]. There is a well-documented pathway for research ranging from observations at the bedside or in the laboratory to smaller-scale trials and, subsequently, large RCTs [14]. In instances where the trials provide strong evidence, the same system can be utilised in an uncontrolled clinical setting in order to generalise the findings and audit the outcomes at the population level.

However, should any variation in outcomes arise, the gold-standard proofing method is to conduct another RCT. Having said that, it is not always feasible, as translating new findings can be even more costly and lead to delays in achieving cost-effectiveness.

### 2.1. Foundations of Combining Research Methodologies

**Figure 1:**
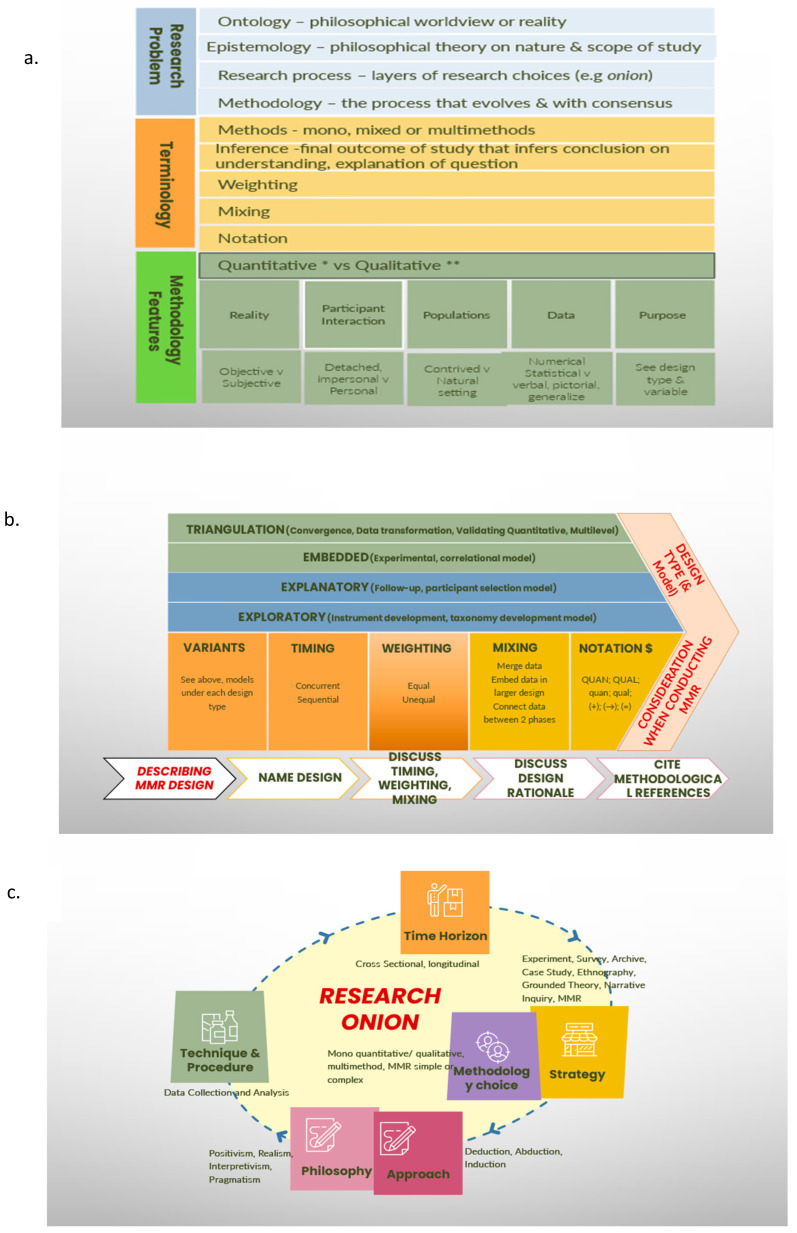
Analysis of research data and determining what information is needed in Phase IV translational work: (**a**) defining the problem, terminology and methodology; (**b**) designing mixed methods research; (**c**) the research onion. Abbreviations: data use = converged data collection; (+) simultaneous; (→) sequential; quan = quantitative; qual = qualitative uppercase = dominant method used, e.g., QUAL; lowercase = lower-priority method, e.g., qual; vs = versus [19] Glossary: http://www.fiu.edu/~bridges/glossary.htm, accessed on 12 November 2023.

It makes sense to incorporate patient experiences when measuring the endpoints and outcomes of treatments being translated. Subjectively, researchers have argued that multimethods and MMR will allow for a more complete understanding of a research problem, with robust quantitative data being integrated with multiple viewpoints [15,16]. How this differs from traditional studies that include smaller studies or quasi-experimental trials is a topic for discussion. Nonetheless, since the 1990s, the distinction of MMR as a methodology has taken shape, as has its use across broad social, educational, behavioural and health sciences fields [17,18]. As is common in evidence-based research, this paradigm took its own course, with debate, skepticism, consensus, and ongoing criticism. As further discussion is not relevant for this paper, the readers are guided accordingly [17,19,20]. Of relevance, however, is the complexity of concepts involved in the conduct of MMR. From the author’s perspective, rigidity is becoming a significant barrier that limits the translation of RCT findings to global populations, and our understanding of MMR is at the heart of this problem. One philosophical assumption argued that MMR is a third research methodology [15]. Thus, for the purpose of the arguments in this paper, we accept the definition of MMR as a combination of different methods (convergent findings, comprehensive coverage, connected contributions) to extract the strengths and reduce the weaknesses of a multimodality approach in a single study [21,22]. Figure 1 highlights the terminology, theories, and a brief historical perspective in the first box; the layers of research in second box; and in the last box, some considerations for formulating MMR studies.

### 2.2. What Are the Issues with Translating Gold Standard Evidence?

**Figure 2:**
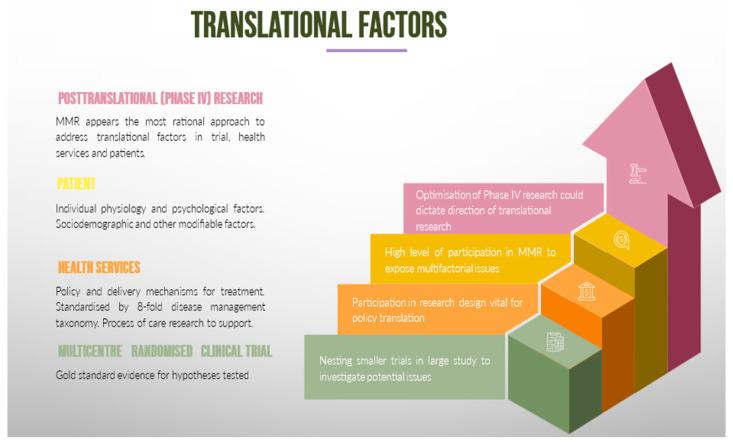
Factors involved in translating trial findings.

The factors at play here can be simplified into three categories: trial, health services and patient factors. Trial related factors are the most established; however, research remains static largely due to the difficulty of conducting tightly controlled studies to remove confounders and match a study group that is closely matched to the treatment arm. This approach has maintained high confidence in the findings and, with that, answering the hypothesis in question. All aspects of the design, methodology, publication, and interpretation of results will in fact reflect this. The strong internal control comes at the expense of reduced generalisability at the population level [23]. Trials, however, need to account for deficiencies in translational outcomes, as post-trial studies explore the health system and patient factors. As existing rigours of drug and treatment discoveries will remain, it will probably be a long while before the right balance can be struck in trial design to meaningfully address these factors. Thus, a greater effort must be made to ensure that post-trial audits are minimised. Secondly, disease management [24] and clinical service organisation studies [25,26] have proven useful in CHF. Hence, these publications have provided proof that organised care is vital for improving CHF outcomes. Thirdly, for patient factors (Figure 2), there are numerous publications of variations in results from CHF trials and the post-trial populations. We will focus the discussion on patient factors below.

### 2.3. Incidental Post-Trial Population-Level Patient Factors in Congestive Heart Failure

The vasodilators used in heart failure studies provided evidence to support the prognostic benefit of nitrate in combination with hydralazine for heart failure with reduced ejection fraction in the 1980s, and they are currently continued as recommended therapies in some circumstances. In the initial study, V-HEFT and V-HEFT1 evidence of a greater benefit in the African American cohort was accumulated. This was then confirmed in the A-HEFT study [27]. Additional studies with ethnographic differentials were noted in the blood pressure trial using diuretics and several renin aldosterone modulating agents, lisinopril and losartan [28,29,30]. Moreover, epidemiological studies in African American cohorts have shown racial differences in the higher incidence of some traditional risk factors, particularly hypertension and modifiable cardiometabolic risk factors [31,32]. Population-based genome-sequencing studies identified differences in alleles’ frequencies with racial-incident HF [33,34]. This extends to black women, for whom higher peripartum cardiomyopathy that is also less responsive to the prescription of angiotensin-converting enzyme inhibitors (ACEi) and β-blockers, was noted [35]. The relative deficiency of natriuretic factors and higher prevalence of salt sensitivity, sodium retention and physiologically low renin and aldosterone levels have treatment implications [36,37,38,39].

From the above physiology, a combination of modifiable and non-modifiable factors must be balanced with acquired and modifiable barriers of socioeconomics in health equity [39]. It is at this juncture that we can start to appreciate the complexity of CHF and achieve equitable outcomes at the population level. Trial controls and trial support either exclude or buffer these differences. However, appreciating that the level of evidence for the proposed question is very high, the opportunity now is to prescribe widely, replicate guidelines and create a mechanism to identify lags in outcomes. If we are to take the example of socio-economic and demography, disparities may not be specific for any one ethnicity. There are multiple levels of influence on racial and socioeconomic disparities in CHF incidence and outcomes [40]. Additionally, models of care to improve these disparities must target layers of sociology, physiology and jurisdictional constraints [41]. Hence, the importance of using subjective and objective data in a single study, as, in these situations, it is now increasingly indispensable [42,43].

## 3. Data Accumulation and Patient Fatigue

An important consideration often not given due consideration, and it is also the focus of this communication, is patient fatigue and dropout. In a phase III randomised controlled trial, patients are the participants. In a post-trial phase IV study, patients can now be considered consumers. Objective data can be obtained from registries or clinical databases and stored as standardised, mostly quantitative performance measures [11]. Within these performance measures are quality measures that include disease management. For the latter, some performance measures will require a subjective (qualitative) research methodology to investigate and then meaningfully inform translational gaps.

### 3.1. Subjective Questionnaires in Medical Research

There is an entire school of knowledge that is required to conduct qualitative research and surveys to obtain patient and customer sentiment, satisfaction, and well-being information [39,40], the purpose of which are as follows:

- Economical and efficient means to collect information, attitudes and opinions from many people or monitor a program’s progress.
- A high level of skill and knowledge is required to design and conduct quality surveys. These skills include knowledge on how to design surveys to answer a focused research question, understanding how to design survey items and response options that will yield interpretable and usable results, understanding survey structures such that the individual items contribute to answers on the research question coherently, and understanding the shortfalls of surveys within the context of the population that they study, including sampling errors, coverage errors, non-response errors, measurement errors, processing errors and transparency indexes [41].
- To understand the ethical and general considerations for the whole spectrum of the population, risk burdens and benefits, vulnerable groups and individuals.
- To be able to draw a conclusion on these subjective points, meaning that surveys must be representative of the population.

The survey response among 904 physicians of varying specialties, surveyed by an established internet-based survey company that offered reminders and gift certificate prizes, was only 35%. Among non-respondents, the main reason for non-participation was lack of time and survey burden [42]. Additionally, 154 of 165 journals (93%) contained minimal guidance on survey reporting, despite 82% of journals publishing qualitative results [44]. There are challenges faced in health services’ qualitative research. The low response rate is a potential contributor to bias, poses challenges in terms of drawing meaningful translational answers and results in a lack of structured guidance to determine the reliability of the data.

### 3.2. Patient Reported Measures and Subjective Questionnaires in Heart Failure

From a report on ‘Crossing the Quality Chasm’, the six domains of health care include safe, timely, efficient, equitable care and patient-centred care, with the latter domain reflecting entirely on topics that the patient is best suited to address [45]. Indeed, patient-reported experiences (PREs) and patient-reported outcomes (PROs) reflect the consumer view on health services and their outcomes. Upon being studied, they are organised into experience and outcome measures, or PROMs and PREMs. There are five dimensions of PROM health-related quality of life (HRQL): functional status, symptoms, symptom burden, health behaviours and patient experience [46].

The definitions of PRO, “as any report of the status of a patient’s health condition that comes directly from the patient, without interpretation of the patient’s response by a clinician or anyone else”, and PROM, as validated questionnaires and tools to extract PROs, are critical the argument on study fatigue [47,48]. Studies can be conducted through several means, including self-completed questionnaires, face-to-face interviews and telephone surveys. Finally, in addition to the six STEEEP (care that is safe, timely, effective, efficient, equitable and patient-centred) factors, we aim for improvement based on core values and guiding principles that place the patient’s interest at the heart of health and social care. Thus patient-centredness requires due consideration of individuality, independence, privacy, partnership, choice, dignity, respect and rights and ethics (beneficence, nonmaleficence, autonomy, and justice). Addressing patient fatigue is, thus, vital for MMR.

### 3.3. Patient Fatigue

In CHF, there are several acknowledged PROM tools that complement clinical, biochemical and imaging biomarkers (Table 1). However, none of these have evolved and are integrated as part of clinical care, despite the compelling reasons for their benefits. Specifically, Kelkar et al. studied a total of 31 instruments: 9 met the inclusion criteria, and only 2 instruments—Minnesota Living with Heart Failure Questionnaire and the Kansas City Cardiomyopathy Questionnaire—met all the evaluation criteria for the psychometric and clinical criteria and symptom coverage [49]. Psotka et al. studied 19 PROMs used in CHF, conducting a review of the characteristics of these instruments’ ability that support potential approvals for the U.S. Food and Drug Administration’s (FDA) product label claim. The Kansas City Cardiomyopathy Questionnaire (KCCQ) and the Minnesota Living with Heart Failure Questionnaire (MLHFQ) had the most extensive evaluation and validation in CHF population. Nonetheless, none of these PROMs met all the relevant criteria listed in the FDA PRO guidance to support product-level claims [50].

The lengths of questionnaires contribute to the response burden and rate. Nonetheless, from the 32 identified and 20 included in the meta-analysis, only 3 studies utilised patients’ input in evaluating response burden. An association between length and response rate was then identified (*p* ≤ 0.0001). As it is not possible to differentiate the impact of content from the length of the questionnaires, not surprisingly, shorter tool lengths have also not been shown to improve this issue [51]. Edwards et al.’s study analyses 38 RCT trials where participants were tasked with completing questionnaires of variable lengths (the actual numbers of pages were unknown). Heterogeneity in regression coefficients in trial response was subsequently explained where meta-regression explained the variations in the length of the questionnaire used in individual trials. For example, in postcard (shortest) questionnaires, the odds of a response more than halved for each extra page (0.39; 95% CI 0.34 to 0.45); for trials of one page in length versus two or three pages, the odds of response for each page increase were 1.01 (95% CI 0.82 to 1.24); for one versus four or more pages, and for two or more pages versus longer questionnaires, the odds ratios per one-page increase were 0.90 (95% CI 0.83 to 0.98) and 0.98 (95% CI 0.96 to 0.99), respectively. Unsurprisingly, it appears that responses can be improved by using shorter questionnaires and with a moderate change in the lengths of short questionnaires, which can be more effective than moderate changes in the lengths of long questionnaires [52]; these findings are supported by similar studies [53,54,55].

### 3.4. Future Areas of Study to Improve Qualitative Research in Heart Failure

Thus far, we have argued the case for MMR to help in the translation of RCT findings, particularly those entrenched in clinical guidelines. In CHF, guidelines reflect a spectrum of care from pharmaceuticals, devices and interventions, as well as allied health comprehensive care. The objective (quantitative) data and its translation are clear. However, despite having robust tools for subjective (qualitative and PRO) data, many steps contribute to study fatigue and lower response rates, and the majority are also not powered sufficiently to inform administrative and regulatory bodies like the FDA in the United States. Strengthening the translational capabilities and using a simpler format should be factors for future discussion. We suggest some broad options:

Questionnaire length—In an 87-patient observational study, Iyngkaran et al. observed chronic disease self-management in CHF. In this study protocol, the authors noted three tiers of subjective data. Clinical interviews and examinations with doctors, NYHA class during each visit, the Flinders Program of Chronic Disease Self-Management, MLWHF-Q, SF-36, PSQ-9 and investigations including 6MWT, echocardiography and biochemistry were used. There was an overlap between objective contributors to disease severity and many of the scales used. The subjective scales ranged from four- to eight-point Likert scales [55,56]. Presently, we have no mechanism to negate the overlapping questions from each tool, factoring in the Likert scale scoring system. The authors suggest a simpler process based on care domains and a no greater than three-point scoring systems and two questions per domain. An example is highlighted in the SCRinHF tool in the study protocol [56].

Translational component—It is important that information gathered through a long trial process carries weight with health administrators and regulators. In this regard, we recommend organizing a focus group to devise the minimum standards required for information in post-trial phase IV studies [56,57,58,59]. Similar to checklists like PRISMA, this simple checklist will ensure that relevant findings will attract relevant health service bodies.

Taxonomy for MMR—Krumholtz et al. provided the impetus for creating a system for disease management. MMR is at the point where overlap in theory and clinical use is sometimes indistinguishable. The theory component for this is strong and grounded in the short history of this methodological process. It is time to create a distinction between research tools, which could use Likert-based broad scales and longer questionnaires, and translational tools, which are more focused on extracting relevant PROs. An important area is the diversity of presentations, even for the same condition, e.g., cognition, rhythm disturbance, ischemic aetiology, etc. [60,61,62,63,64,65,66,67]. The simplification of established tools will also require validation. Prior to this, there must be an agreement on the direction that will be taken in this process. This will probably become a subject of greater debate in time.

**Table 1:** Qualitative tools measuring outcomes for HF and self-care programs.

Tool	Type of Measure	Summary of Instrument/Tool	Dimensions
**ACIC**	Health Systems	The components of ACIC were derived after specific evidence-based interventions from the six components of the Chronic Care Model. Thus, similar to this model, the ACIC addresses the main elements for improving chronic illness care at the community, organisation, practice and patient levels.	Many measures were considered: Community resources; Health organisation; Self-management support; Delivery system design; Decision support; Clinical information systems.
**PACIC**	Patient Satisfaction	20- or 26-item patient report instruments were used to rate chronic illness care over a 6-month period. They cover 5 dimensions of care.	Many measures were considered: Patient activation; Delivery system design; Goal setting; Problem solving; Follow-up/coordination.
**PSQ-18**	Patient satisfaction	Short form of PSQ-III using a Likert scale questionnaire evaluating 18 items from 7 dimensions of patient satisfaction directed toward doctors.	Many measures were considered: General satisfaction; Technical quality; Interpersonal manner; Communication; Financial aspects; Time spent with doctor; Accessibility and convenience.
**CAHPS**	Patient satisfaction	Survey for consumers and patients to report on and evaluate their experiences with health care across 12 dimensions.	Many measures were considered: Getting timely care; Provider communication; Rating of provider; Access to specialists; Health promotion and education; Shared decision-making; Health status/Functional Status; Courteous/helpful office staff; Care coordination; Between-visit communication; Education about medication adherence; Stewardship of patient resources.
**SF-36v2**	Patient reported outcomes	Patient-reported 5-point survey covering mental and physical health over eight scaled scores. Each question has equal weight, with final score from 0 to 100 scale. Lower scores are associated with greater disability.	Many measures were considered: Physical functioning; Physical role functioning; Bodily pain; General health perceptions; Vitality; Emotional role functioning; Social role functioning; Mental health.
**EQ-5D**	Patient reported outcomes	The most used self-administered survey, being available in >70 languages, that can be completed within minutes. Scoring based on a 3-point descriptive questionnaire and 20 cm vertical visual analogue scale with best (top) or worst health (bottom).	Many measures were considered: Mobility; Self-care; Usual activities; Pain/discomfort; Anxiety/depression.
**QWB-SA**	Patient reported outcomes	Survey of an interview with 71 items scored from 0 (death) to 1.0 (full function) taking 10–15 min. It can be translated into QALY. It requires training.	Many measures were considered: Acute and chronic symptoms; Self-care; Mobility; Physical activity; Usual activity.
**HUI**	Patient reported outcomes	A family of generic health profiles and preference-based systems measuring health status, reporting health-related quality of life, and producing utility scores. It explores the following: (1) experience of patients undergoing therapy; (2) long-term outcomes of disease or therapy; (3) the efficacy, effectiveness, and efficiency of interventions; and (4) health status of general populations. Each HUI attribute (dimension) has 3–6 levels of discrimination and is very responsive to changes in health caused by treatment therapies or other influences.	Three measures were considered: 8 attributes vision, hearing, and speech; Ambulation, dexterity, and emotion; Cognition and pain—each with 5 or 6 levels of ability/disability.
**KCCQ**	Disease specific QOL	The Kansas City Cardiomyopathy Questionnaire (KCCQ) is a new, self-administered, 23-item questionnaire developed to provide a better description of HRQoL in patients with CHF. It quantifies, in a disease-specific fashion, physical limitation, symptoms (frequency, severity and recent change over time), QoL, social interference, and self-efficacy.	Many measures were considered: Physical limitations; Symptoms’ stability and frequency, severity, and change over time; Self-efficacy and knowledge; Social interference/limitation; QoL.
**MLHFQ**	Disease specific QOL	A self-administered, 5–10 min, 21-item 5-point Likert variable used to measure the effects of symptoms, functional limitations, and psychological distress on an individual’s quality of life, the MLHF questionnaire asks each person to indicate using a 6-point, zero to five, Likert scale on how much each of 21 facets prevented them from living as they desired. The MLHFQ is designed to measure the effects of heart failure and its treatments on an individual’s quality of life. MLHFQ measures the effects of symptoms, functional limitations, and psychological distress on an individual’s quality of life. It consists of questions that assess the impacts of frequent physical symptoms, the effects of heart failure on physical/social functions, and side effects of treatments, hospital stays, and costs of care.	
**NYHA**	Disease specific QOL	A standardised health care provider assessment of heart failure severity. Dyspnoea grading with varying states of rest and exercise. Range of 0–4. Higher scores are worse.	One component—Universal.
**CFPI**	Understanding self-care and goals	Partners in Health Scale tests self-efficacy for managing chronic disease using a 6-item scale, Energy/Fatigue Scale, Cue and Response Score, and Problems and Goals Score. Training required for use.	Three testing methods were used: Partners in Health; Cue and Response Score; Problems and Goals Score.
**EHFScBS**	CHF self-care	The EHFScBS is a 12-item questionnaire that measures 3 aspects of health maintenance behaviours: compliance with their management regimen, asking for help, and adapting daily activities. Responses are on a 5-point Likert-type scale indicating how often each behaviour is performed, ranging from “I completely agree” to “I don’t agree at all”. Scores are summed. Lower scores indicate better self-care. The instrument has subsequently been revised into a 9-item instrument.	Translated into 14 languages: Swedish (161); The Netherlands (1243); United Kingdom (177); Italian (173); German (285); Spanish (553).
**SCHFI**	CHF self-care	The SCHFI consists of 15 items that measure 3 subscales: behaviours undertaken to maintain clinical stability (self-care maintenance), the decision-making process with regard to symptom changes (self-care management), and the confidence to manage symptoms and evaluate any actions implemented (self-care confidence). Self-care management can only be computed if patients have been symptomatic in the past month. Summary scores for the 3 subscales are used by transforming each subscale into a scale from 0 to 100. Adequate scores are more than 70 on any subscale.	Officially translated into Spanish and Thai languages and requests to use it in 24 other countries: United States (453); Australian (1095); Thai (400); Mexican (134).

2DE, BNP and 6MWT are simple reproducible qualitative tools that can be combined with routine biochemistry. Abbreviations: CAHPS—Consumer Assessment of Healthcare Providers and Systems; EQ-5D—EuroQOL five dimensions questionnaire; HUI—health utility index; KCCQ—Kansas City Cardiomyopathy Questionnaire; MLHFQ—Minnesota Living with Heart Failure questionnaire; PACIC—Patient Assessment of Chronic Illness Care; PSQ-18—The Patient Satisfaction Questionnaire Short Form; PRO—patient reported outcomes; QOL—quality of life; QWB-SA—quality of well-being self-administered version. Details of table compiled from reference [59].

## 4. Conclusions

Although research can be complex, the arguments in this paper are in favour of simplicity. In a sense, to accumulate new knowledge in an area warrants multiple dimension framework and protocols. To provide the solution to the lay public, it must be contextual. In this paper, we have not fully explored those details as our focus was to argue that the issues of patient burden and fatigue are real. Nonetheless, there are foundations for how PROs are designed and used. It will often be the case that the early steps create a complex tool. In the latter steps, we require a case-by-case scenario to address these needs. In time, we believe that guidelines could evolve to help to steer this area. Presently, we are not aware of a consensus statement or approach to meet this goal. Some broad guidelines conclude, given the inherently problematic nature of comparing questionnaires of various lengths, that it is preferable to base decisions regarding the use of instruments on the content rather than the length per se. If a choice of follow-up questionnaire exists for a clinical trial, the shorter one should be used. If a new follow-up questionnaire is to be designed, it should be made as short as possible, without compromising the data collection requirements of the trial. This field is still evolving, and additional exploration and brainstorming will be necessary to progress positively.

## Data Availability

Data used in this perspective were publically available and most information provided here are from references listed in this paper.
